# Immune Microenvironment Heterogeneity in Breast Cancer: Stromal Lymphocytic Patterns Across Molecular and Clinicopathologic Subtypes

**DOI:** 10.3390/diagnostics16213454

**Published:** 2026-10-22

**Authors:** Mariam Shadan, Nazoora Khan, Mohammad Amanullah Khan

**Affiliations:** 1 College of Medicine, Dubai Medical University, Dubai 19099, United Arab Emirates; 2 Jawaharlal Nehru Medical College, Aligarh Muslim University, Aligarh 202001, India

**Keywords:** tumour-infiltrating lymphocytes, stromal lymphocytic infiltration, breast cancer, molecular subtype, immune microenvironment, histopathology

## Abstract

***Background/Objectives***: Tumour-infiltrating lymphocytes (TILs) have emerged as important indicators of tumour–host immune interaction in breast cancer, with increasing prognostic and predictive significance across molecular subtypes. However, most available evidence is derived from Western populations and focuses predominantly on quantitative stromal TIL assessment, while data regarding morphologic stromal immune architecture in Asian populations remain limited. This study evaluated stromal lymphocytic infiltration patterns and their association with molecular and clinicopathologic characteristics in an Indian cohort of invasive ductal carcinoma of the breast. ***Methods***: This retrospective observational pathology-based study included 231 female patients diagnosed with invasive ductal carcinoma at Jawaharlal Nehru Medical College, Aligarh Muslim University, India, between 2009 and 2014. Stromal lymphocytic infiltration was assessed on haematoxylin and eosin-stained sections using a semi-quantitative four-tier scoring system adapted from Guo et al. with whole-section assessment at ×100 magnification by two pathologists. Associations with tumour grade, tumour size, lymph node status, and molecular subtype were assessed using exact and ordinal tests with Holm correction for multiple comparisons. ***Results***: Luminal breast carcinoma was the most common molecular subtype (59.3%), followed by HER2-positive tumours (22.5%) and triple-negative breast cancer (18.2%). Sparse stromal lymphocytic infiltration (score 1) represented the most frequent pattern overall (46.8%). Significant associations were identified between stromal lymphocytic infiltration and molecular subtype, tumour grade, and lymph node status (Holm-adjusted *p* < 0.001, *p* = 0.044 and *p* = 0.044, respectively). Triple-negative and HER2-positive tumours demonstrated higher proportions of moderate-to-dense (score 2–3) infiltration than luminal tumours (40.5% and 48.1% vs. 16.8%). Score 2–3 infiltration was more frequent in Grade III tumours (51.2%) and, conversely, in lymph node-negative than in lymph node-positive tumours (40.8% vs. 21.9%). No statistically significant association was observed between stromal lymphocytic infiltration and tumour size (Holm-adjusted *p* = 0.071). ***Conclusions***: Stromal lymphocytic infiltration patterns demonstrate substantial heterogeneity across breast cancer molecular subtypes and are associated with molecular subtype and histologic grade, whereas higher infiltration was more frequent in lymph node-negative tumours. These descriptive associations suggest that stromal immune patterns vary with tumour phenotype rather than tumour size. Semi-quantitative morphologic stromal lymphocyte assessment on routine histopathology is inexpensive and feasible in resource-limited settings; its reproducibility and prognostic value require validation.

## 1. Introduction

Breast cancer is a biologically heterogeneous malignancy in which tumour behaviour is influenced not only by molecular subtype, but also by the composition and activity of the tumour microenvironment [1]. Among the various components of the tumour microenvironment, tumour-infiltrating lymphocytes (TILs) have emerged as important mediators of tumour–host immune interaction and increasingly relevant prognostic and predictive biomarkers in breast cancer [2]. Stromal TILs, composed predominantly of cytotoxic T lymphocytes, helper T cells, regulatory T cells, and B lymphocytes, are considered more reproducible for routine histopathological assessment than intratumoral lymphocytes [1,3]. Consequently, the International TIL Working Group recommended standardised evaluation of stromal TILs on haematoxylin and eosin-stained sections to improve reproducibility and facilitate integration of TIL assessment into routine breast pathology practice [4]. High stromal lymphocytic infiltration has consistently been associated with improved therapeutic responsiveness and favourable outcomes in triple-negative and HER2-positive breast cancers, reflecting the enhanced immunogenicity of these biologically aggressive subtypes [2,5,6]. In contrast, the biologic significance of stromal lymphocytic infiltration in hormone receptor-positive breast cancer remains less clearly defined and appears to demonstrate substantial molecular and clinicopathologic heterogeneity [7,8].

Despite increasing interest in TILs, several challenges continue to limit their broader clinical application. Most available evidence originates from Western populations and predominantly focuses on triple-negative and HER2-positive disease, whereas data from Asian and low-resource settings remain comparatively limited [9,10]. Furthermore, while percentage-based stromal TIL assessment provides quantitative estimation of immune infiltration, it may incompletely characterise morphologic stromal immune architecture, including lymphoid organisation and follicle formation. Recent studies from Asian populations have demonstrated that stromal lymphocytic infiltration correlates strongly with aggressive clinicopathologic features such as high tumour grade, elevated proliferative activity, and adverse molecular subtypes, suggesting possible regional and biologic variability in tumour immune landscapes [9,10]. These observations underscore the need for practical and reproducible histo-morphologic approaches capable of evaluating broader stromal immune patterns in routine diagnostic pathology.

Therefore, the present study aimed to evaluate stromal lymphocytic infiltration patterns in invasive ductal carcinoma of the breast using a semi-quantitative stromal scoring system adapted from Guo et al. [11] and to determine their association with molecular subtype and established clinicopathologic prognostic markers in an Indian cohort of treatment-naïve breast cancer patients. Specifically, this study sought to answer the following research questions: (1) Are stromal lymphocytic infiltration patterns associated with established clinicopathologic prognostic factors in breast cancer? (2) Do stromal lymphocytic patterns vary across molecular subtypes of breast carcinoma? (3) Can routine histo-morphologic assessment of stromal lymphocytes reflect underlying immune microenvironment heterogeneity in breast cancer?

## 2. Materials and Methods

### 2.1. Study Design and Setting

This retrospective observational pathology-based study was conducted at the Department of Pathology, Jawaharlal Nehru Medical College, Aligarh Muslim University, India. The study evaluated histopathological specimens from female patients diagnosed with invasive ductal carcinoma (IDC) of the breast. All breast carcinoma cases diagnosed in the department between 2009 and 2014 were identified from the departmental archive and screened for eligibility. Histopathological review, stromal lymphocyte scoring and data analysis were performed on the archived material after institutional ethics approval had been obtained (27 July 2017). All specimen types received in the department were eligible, including core needle biopsies, excision biopsies/lumpectomies and mastectomy specimens.

### 2.2. Study Population

**Figure 1:**
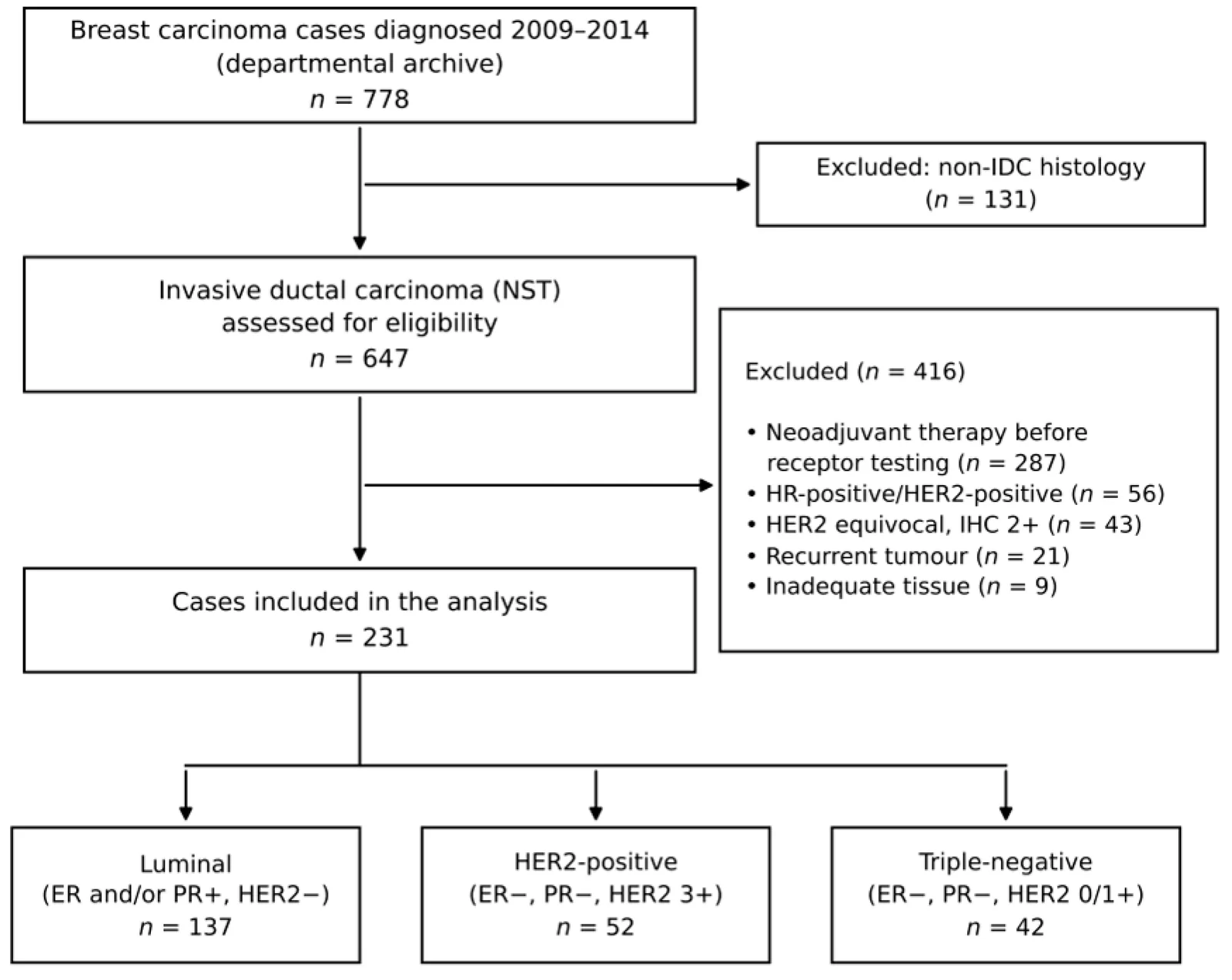
Flow diagram of case selection. All cases were retrieved from the departmental archive. HR, hormone receptor; IDC, invasive ductal carcinoma; IHC, immunohistochemistry; NST, no special type.

A total of 231 histopathologically confirmed cases of invasive ductal carcinoma were included in the study. Inclusion criteria were: (i) female patients; (ii) histopathologically confirmed primary invasive ductal carcinoma (invasive breast carcinoma of no special type); (iii) ER, PR and HER2 immunohistochemistry performed on tissue obtained before any neoadjuvant systemic therapy or radiotherapy; and (iv) availability of adequate haematoxylin and eosin (H&E)-stained sections containing invasive tumour with intervening stroma. Exclusion criteria were: (i) receipt of neoadjuvant therapy before the tissue used for receptor testing was obtained, including patients treated at other centres before referral; (ii) recurrent tumours; (iii) non-IDC histology; (iv) HER2-equivocal (IHC 2+) tumours; (v) tumours co-expressing hormone receptors and HER2 (hormone receptor-positive/HER2-positive, “triple-positive”); and (vi) inadequate or poorly preserved tissue. Of 778 breast carcinoma cases screened, 131 with non-IDC histology were excluded; of the remaining 647 IDC cases, a further 416 were excluded (neoadjuvant therapy before receptor testing, *n* = 287; HR-positive/HER2-positive tumours, *n* = 56; HER2-equivocal tumours, *n* = 43; recurrent tumours, *n* = 21; inadequate tissue, *n* = 9), leaving 231 cases for analysis. The case selection process is summarised in Figure 1.

### 2.3. Clinicopathologic Data

Clinicopathologic parameters retrieved from pathology records included patient age, tumour size, histologic grade, lymph node status, and immunohistochemical expression of estrogen receptor (ER), progesterone receptor (PR), and HER2/neu. Histologic grade was assigned using the Nottingham (Elston–Ellis modification of the Scarff–Bloom–Richardson) system recommended by the WHO classification, and tumour size was categorised according to the TNM pT classification (T1, ≤20 mm; T2, >20–50 mm; T3, >50 mm). ER and PR were scored using the Allred system, and tumours with an Allred score ≥3 were considered positive. HER2 was scored according to the 2013 ASCO/CAP guideline: scores of 0 and 1+ were considered negative and 3+ (complete, intense circumferential membrane staining in >10% of tumour cells) was considered positive; HER2-equivocal (2+) tumours were excluded because confirmatory in situ hybridisation was not available. Tumours were classified as luminal (ER- and/or PR-positive, HER2-negative), HER2-positive (ER- and PR-negative, HER2-positive) or triple-negative (ER-, PR- and HER2-negative). Because Ki-67 was not available, luminal A and luminal B tumours could not be distinguished and are reported as a single luminal group.

### 2.4. Histopathologic Assessment of Stromal Lymphocytes

Haematoxylin and eosin-stained tumour sections were reviewed independently for stromal lymphocytic infiltration using a semi-quantitative scoring system adapted from Guo et al. [11]. This pattern-based system was chosen, rather than the percentage-based stromal TIL assessment recommended by the International TIL Working Group [4], because the aim was to record the architectural organisation of the stromal infiltrate, including lymphoid follicle formation, which a continuous percentage does not capture. Guo et al. defined the categories qualitatively [11]; the operational criteria applied in the present study are given in parentheses. Stromal lymphocytic infiltration was graded as follows:

- Score 0: No lymphocytes in the stroma;
- Score 1: Few lymphocytes in the stroma (scattered, isolated lymphocytes or small loose clusters occupying only a minor proportion of the stromal area);
- Score 2: Abundant stromal lymphocytes without lymphoid follicle formation (dense, confluent lymphocytic infiltrate occupying a substantial proportion of the stromal area, without circumscribed follicles);
- Score 3: Abundant stromal lymphocytes with lymphoid follicle formation (as for score 2, with at least one lymphoid follicle).

The assessment focused on stromal lymphocytes surrounding invasive tumour nests, excluding areas of necrosis and crush artefact. Lymphocytes within tumour nests, in normal breast lobules or ductal carcinoma in situ, and in areas of biopsy-site change were not scored. All available H&E sections of each case were examined in their entirety at ×100 magnification (10× objective), as the score describes the overall pattern of infiltration rather than a cell count; therefore, no fixed number of fields was used, and each case was assigned a single overall score according to the criteria of Guo et al. [11]. A lymphoid follicle was defined as a well-circumscribed, rounded, nodular aggregate of densely packed small lymphocytes within the tumour stroma; the follicles observed were primary follicles without germinal centres. Diffuse sheets or ill-defined dense aggregates without this circumscribed nodular organisation were scored as 2. Each case was scored independently by two pathologists who were blinded to receptor status and clinicopathologic data; discordant scores were resolved by a third pathologist. Interobserver agreement was not formally quantified, and percentage-based stromal TIL values were not recorded. Representative photomicrographs were captured using a smartphone camera held at the eyepiece of a conventional light microscope with the 10× objective; because no calibrated imaging system was available, spatial calibration was not possible and scale bars could not be added.

### 2.5. Statistical Analysis

Data were analysed using SPSS software version 21. Categorical data are presented as counts with row percentages. Associations between the stromal lymphocyte score and each clinicopathologic variable were assessed using Pearson’s chi-square test; because some cells had expected counts below 5 (minimum 3.1), exact *p*-values were obtained using the Fisher–Freeman–Halton test (Monte Carlo estimate, 50,000 samples), and effect size was expressed as Cramér’s V. As the stromal score is ordinal, ordered associations with tumour size, histologic grade and lymph node status were additionally tested using the linear-by-linear association test. Differences between molecular subtypes in the proportion of moderate-to-dense infiltration (scores 2–3 vs. 0–1) were compared pairwise using Fisher’s exact test. The Holm–Bonferroni method was used to correct for the four primary comparisons and for the three pairwise subtype comparisons. A two-sided adjusted *p* < 0.05 was considered statistically significant. Unless otherwise stated, *p*-values reported in the text are Holm-adjusted; Table 1 reports both unadjusted and Holm-adjusted exact *p*-values, and the linear-by-linear trend tests are reported as unadjusted secondary analyses. Exact and ordinal tests were performed in Python (SciPy version 1.18).

## 3. Results

A total of 231 female patients diagnosed with invasive ductal carcinoma of the breast were included in the study. Of 778 breast carcinoma cases screened, 547 were excluded (Figure 1). The majority of patients belonged to the 45–60 years age group, accounting for 43.8% of the cohort, followed by patients aged less than 45 years (39.5%). Patients older than 60 years represented the smallest subgroup (16.7%). Molecular subtype analysis demonstrated that luminal breast carcinoma was the most common subtype, comprising 137 cases (59.3%), followed by HER2-positive tumours in 52 cases (22.5%) and triple-negative breast cancer (TNBC) in 42 cases (18.2%).

**Figure 2:**
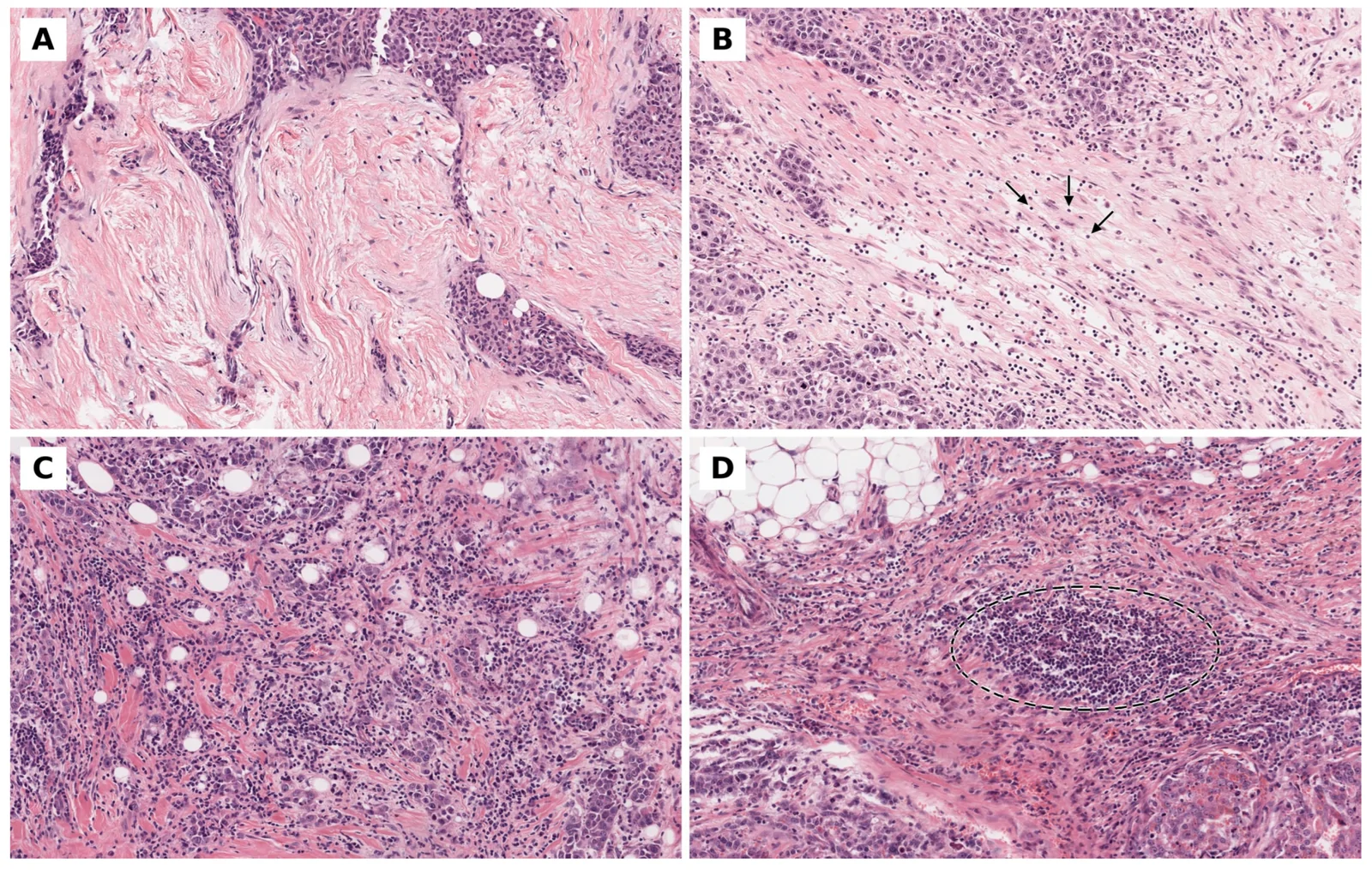
Representative histopathological patterns of stromal lymphocytic infiltration in invasive ductal carcinoma of the breast. (**A**) Score 0: absence of stromal lymphocytes. (**B**) Score 1: sparse stromal lymphocytic infiltration (arrows indicate representative stromal lymphocytes). (**C**) Score 2: abundant stromal lymphocytic infiltration without lymphoid follicle formation. (**D**) Score 3: dense stromal lymphocytic infiltration with lymphoid follicle formation (dashed outline). (Haematoxylin and eosin stain; all panels photographed with the 10× objective [original magnification ×100] using a smartphone camera at the eyepiece; scale bars could not be added because the images were not spatially calibrated.)

Stromal lymphocytic infiltration was assessed using a semi-quantitative four-tier scoring system adapted from Guo et al. [11]. Representative histopathological patterns corresponding to the four stromal lymphocyte scores are illustrated in Figure 2. Sparse stromal lymphocytic infiltration predominated within the cohort, with score 1 representing the most frequently observed pattern, identified in 108 cases (46.8%). Complete absence of stromal lymphocytes (score 0) was observed in 58 cases (25.1%). More pronounced stromal immune infiltration patterns were less frequent, with score 2 observed in 48 cases (20.8%) and score 3, characterised by abundant stromal lymphocytic infiltration with lymphoid follicle formation, identified in 17 cases (7.4%).

The association between stromal lymphocytic infiltration patterns and clinicopathologic variables is summarized in Table 1. A statistically significant association was identified between stromal lymphocytic infiltration and molecular subtype classification (Holm-adjusted *p* < 0.001; Cramér’s V = 0.29). Luminal tumours predominantly demonstrated sparse stromal lymphocytic infiltration, with score 1 representing the most common pattern, observed in 82 of 137 cases, whereas only five luminal tumours demonstrated score 3 infiltration. In contrast, TNBC demonstrated comparatively greater stromal immune infiltration, with 17 of 42 tumours exhibiting scores 2–3, including eight tumours demonstrating organised lymphoid follicle formation; however, TNBC was heterogeneous, as 15 of 42 tumours (35.7%) lacked stromal lymphocytes (score 0). HER2-positive tumours similarly exhibited enhanced stromal immune infiltration, with score 2 representing the predominant infiltration pattern in this subgroup, identified in 21 of 52 cases. The proportion of score 2–3 infiltration was higher in TNBC- (40.5%) and HER2-positive tumours (48.1%) than in luminal tumours (16.8%; Holm-adjusted *p* = 0.005 and *p* < 0.001, respectively), with no difference between TNBC- and HER2-positive tumours (Holm-adjusted *p* = 0.53).

Tumour grade also demonstrated a significant association with stromal lymphocytic infiltration patterns (Holm-adjusted *p* = 0.044 [unadjusted exact *p* = 0.015]; Cramér’s V = 0.19; unadjusted linear-by-linear *p* = 0.004). Grade I tumours largely exhibited sparse stromal immune infiltration, while Grade III tumours demonstrated comparatively greater stromal lymphocytic infiltration, with more than half of Grade III tumours exhibiting scores 2–3 (22 of 43; 51.2%). In contrast, the proportions of score 2–3 infiltration were similar in Grade I (23.8%) and Grade II (21.7%) tumours, indicating that the association was driven mainly by Grade III tumours.

A statistically significant association was additionally observed between stromal lymphocytic infiltration and lymph node status (Holm-adjusted *p* = 0.044 [unadjusted exact *p* = 0.015]; Cramér’s V = 0.21; unadjusted linear-by-linear *p* = 0.006). Higher infiltration was associated with lymph node negativity: scores 2–3 were observed in 31 of 76 (40.8%) lymph node-negative tumours compared with 34 of 155 (21.9%) lymph node-positive tumours, whereas absent or sparse infiltration (scores 0–1) predominated among node-positive tumours (121 of 155; 78.1%).

No statistically significant association was observed between stromal lymphocytic infiltration and tumour size (Holm-adjusted *p* = 0.071). Across all tumour size categories, sparse stromal lymphocytic infiltration (score 1) remained the predominant pattern. Although larger tumours demonstrated relatively higher proportions of pronounced stromal immune infiltration (score 2–3 in 36.0% of T3 vs. 25.2% of T1 tumours), these differences did not achieve statistical significance. T3 tumours also showed the highest proportion of absent infiltration (score 0, 36.0%).

Overall, higher stromal lymphocytic infiltration patterns were more frequently associated with biologically aggressive clinicopathologic features, namely Grade III histology and non-luminal molecular subtypes, whereas, conversely, higher infiltration was more frequent in lymph node-negative tumours. TNBC- and HER2-positive tumours demonstrated comparatively greater stromal immune infiltration than luminal tumours, whereas stromal lymphocytic infiltration did not demonstrate a significant association with primary tumour size.

## 4. Discussion

The present study evaluated stromal lymphocytic infiltration patterns in treatment-naïve invasive ductal carcinoma of the breast and demonstrated significant associations between stromal immune architecture and several established clinicopathologic prognostic markers. Higher stromal lymphocytic infiltration patterns were more frequently observed in biologically aggressive tumours, particularly high-grade carcinomas, HER2-positive tumours, and triple-negative breast cancers, whereas luminal tumours predominantly demonstrated sparse stromal immune infiltration. Higher stromal infiltration was more frequent in lymph node-negative than in node-positive tumours, although no significant relationship was identified with primary tumour size. Collectively, these descriptive findings indicate that stromal lymphocytic patterns vary with tumour phenotype rather than tumour size and illustrate the substantial heterogeneity of the breast cancer immune microenvironment across molecular subtypes.

The enrichment of stromal lymphocytic infiltration within HER2-positive and triple-negative tumours observed in the present study is consistent with current understanding of breast cancer immunogenicity. Several studies have demonstrated that TNBC- and HER2-positive breast cancers possess higher mutational burden, greater genomic instability, and increased neoantigen expression, thereby generating stronger host immune responses than luminal tumours [1,2,3]. Denkert et al. [5] demonstrated that elevated stromal TIL levels in TNBC- and HER2-positive tumours correlated strongly with pathological complete response following neoadjuvant chemotherapy. Similarly, Loi et al. [6] reported that increased stromal TIL density in TNBC was associated with improved disease-free and overall survival, further highlighting the biologic relevance of immune infiltration in aggressive breast cancer subtypes. Our findings closely parallel those reported by Vaid et al. [9] in an Indian cohort and Duong et al. [10] in a Vietnamese population, both of which demonstrated significantly higher stromal immune infiltration in TNBC compared with hormone receptor-positive tumours. Together, these studies indicate that subtype-related differences in stromal infiltration are also observed in Asian populations. Notably, TNBC in our cohort was heterogeneous, with more than one-third of tumours lacking stromal lymphocytes, consistent with the existence of immunologically distinct TNBC subsets.

An important observation in the present study was the association between stromal lymphocytic infiltration and histologic grade. Grade III tumours showed a higher proportion of moderate-to-dense infiltration than lower-grade tumours, whereas Grade I and II tumours were similar, a finding repeatedly described in previous studies [10,12,13]. Baek et al. [12] demonstrated that high stromal TIL levels in ER-positive/HER2-negative tumours were strongly associated with histologic Grade III disease and increased pathological complete response following neoadjuvant chemotherapy. Similarly, Kim and Koo [13] showed that immune-related markers such as CD8, FOXP3, CD68, and CD163 were significantly enriched in high-grade and HER2-enriched tumours. One possible explanation, which the present study could not test directly, is that poorly differentiated tumours elicit stronger immune recruitment through greater neoantigen generation.

In contrast to grade and molecular subtype, higher stromal lymphocytic infiltration was more frequent in lymph node-negative tumours (40.8% vs. 21.9% with scores 2–3). This inverse relationship is consistent with the favourable prognostic associations of higher stromal TIL levels reported in large cohorts and meta-analyses [2,5,6] and may indicate that a pronounced stromal immune response accompanies more limited regional spread. It contrasts with the findings of Guo et al. [11], who used the same scoring categories in invasive micropapillary carcinoma and observed more frequent nodal metastasis in tumours with abundant stromal lymphocytes, and with those of Criscitiello et al. [7], who reported an association between stromal TILs and nodal involvement in ER-positive/HER2-negative tumours, suggesting that this relationship may differ between histologic and molecular subtypes. Because nodal status is related to both grade and molecular subtype and multivariable adjustment was not possible, this association should be regarded as hypothesis-generating.

Interestingly, no statistically significant association was identified between stromal lymphocytic infiltration and primary tumour size. This suggests that the extent of stromal infiltration is not simply proportional to tumour bulk, although the borderline *p*-value (0.071) indicates that a modest relationship cannot be excluded in a larger cohort. Similar observations have been reported in previous studies where stromal TIL enrichment correlated more strongly with molecular subtype, proliferative activity, and histologic grade than with tumour dimensions [9,10].

The predominantly sparse stromal immune infiltration observed in luminal tumours within the present study is similarly consistent with the traditionally “immune-cold” microenvironment associated with hormone receptor-positive breast cancer. Prior studies have consistently demonstrated lower stromal TIL density in luminal tumours compared with HER2-positive and TNBC subtypes [7,8]. Nevertheless, the biologic significance of immune infiltration within luminal disease remains considerably more complex than in other molecular subtypes. Pousette et al. [8] demonstrated that while elevated stromal TIL levels in ER-positive/HER2-negative tumours correlated with poor prognostic clinicopathologic variables, specific immune-related gene signatures retained independent prognostic significance. Similarly, Criscitiello et al. [7] observed that elevated stromal TIL levels were associated predominantly with luminal B tumours and increased proliferative activity. In the present study, luminal tumours generally showed sparse stromal infiltration; however, because Ki-67 was not available, luminal A and B tumours could not be separated, and we could not examine whether higher infiltration in luminal tumours was concentrated in luminal B-like tumours.

One of the distinctive aspects of the present study is the use of a semi-quantitative morphologic scoring system adapted from Guo et al. [11], rather than the percentage-based stromal TIL assessment recommended by the International TIL Working Group [4]. While percentage-based TIL assessment provides standardisation and facilitates comparison across studies, it may incompletely characterise broader stromal immune architecture, particularly lymphoid organisation and follicle formation. Our scoring approach additionally captured morphologic patterns of organised stromal lymphoid aggregates, in the form of lymphoid follicles, which were most frequent in TNBC (19.0%) compared with HER2-positive (7.7%) and luminal tumours (3.6%). We have deliberately described these as lymphoid follicles rather than tertiary lymphoid structures (TLSs): although organised lymphoid aggregates in tumours may correspond to TLSs, which have been associated with active local immune responses [1,3], confirmation of TLSs requires immunohistochemical demonstration of segregated B- and T-cell zones, follicular dendritic cell networks and high endothelial venules, which was not performed. Whether such architectural information adds to quantitative stromal TIL density remains to be established, as percentage-based stromal TILs were not recorded in parallel.

The findings of this study may also have practical relevance for routine pathology practice, particularly in low-resource and low- and middle-income settings. Advanced molecular immune profiling, multiplex immunohistochemistry, and AI-assisted digital pathology remain inaccessible in many institutions because of infrastructural and economic limitations. In contrast, semi-quantitative stromal lymphocytic assessment on routine haematoxylin and eosin-stained sections represents a practical, inexpensive, and widely applicable method for evaluating tumour immune microenvironment patterns. This is especially relevant in South Asian populations, where younger age at presentation, aggressive molecular subtypes, and higher TNBC prevalence are frequently encountered [9]. Whether such assessment can contribute to risk stratification is, however, a hypothesis that requires validation in cohorts with outcome data.

Several limitations should nevertheless be acknowledged. First, the study was retrospective, and survival and treatment-response data were not available; therefore, neither causal nor prognostic inferences can be drawn, and any diagnostic or prognostic application of the score remains a hypothesis. Second, all analyses were univariable; because tumour grade, molecular subtype and nodal status are interrelated, the reported associations may be confounded, and multivariable analysis in a larger cohort with individual-level data is required. Third, the scoring system is semi-quantitative and morphological; it has not been validated against the percentage-based stromal TIL method of the International TIL Working Group [4], percentage stromal TILs were not recorded in parallel, and interobserver agreement was not formally quantified, which limits reproducibility and comparison with other studies. Fourth, all specimen types, including small biopsies, were included; because lymphocytic infiltration may be spatially heterogeneous, biopsies may not fully represent the stromal infiltrate of the whole tumour. Fifth, lymphoid follicles were identified on H&E morphology alone and were not characterised immunohistochemically; they therefore cannot be equated with tertiary lymphoid structures. Sixth, Ki-67 was not available, so luminal A and B tumours could not be distinguished, and the exclusion of HER2-equivocal and hormone receptor-positive/HER2-positive tumours limits generalisability to these groups. Finally, this was a single-centre study, and some subgroups (e.g., score 3 tumours) were small. Additionally, immunophenotypic characterisation of lymphocyte subpopulations was not performed; therefore, the functional composition of stromal infiltrates could not be determined. Given the increasing importance of immune cell subtype profiling, future studies incorporating CD8, FOXP3, PD-L1, and macrophage markers may provide deeper mechanistic insight into subtype-specific immune microenvironment behaviour. Furthermore, emerging advances in digital pathology and artificial intelligence-based TIL quantification may improve reproducibility and standardization of stromal immune assessment in future studies [14].

## 5. Conclusions

The present study demonstrates that stromal lymphocytic infiltration patterns are associated with molecular subtype and histologic grade in invasive ductal carcinoma of the breast. Higher stromal immune infiltration was more frequently observed in triple-negative, HER2-positive, and Grade III tumours and, conversely, in lymph node-negative rather than lymph node-positive tumours, whereas luminal tumours predominantly exhibited sparse stromal lymphocytic patterns. These descriptive associations indicate that stromal immune patterns vary with tumour phenotype rather than tumour size. Routine morphological assessment of stromal lymphocytes is inexpensive and feasible in resource-limited settings; however, its reproducibility, its relationship to standardised stromal TIL assessment and its prognostic or predictive value remain to be validated. Future studies incorporating multivariable and survival analysis, immunophenotypic characterisation, and standardized digital pathology approaches may further clarify the prognostic and therapeutic significance of stromal immune architecture in breast cancer.

## Figures and Tables

**Table 1:** Association between stromal lymphocytic infiltration patterns and clinicopathologic characteristics in invasive ductal carcinoma of the breast.

Parameter	Category	n	Stromal Lymphocyte Score [11], n (%)				Exact *p*-Value: Unadjusted/Holm-Adjusted (Cramér’s V)
			0	1	2	3	
Tumour size	T1	107	27 (25.2)	53 (49.5)	20 (18.7)	7 (6.5)	0.071/0.071 (0.16)
	T2	74	13 (17.6)	41 (55.4)	16 (21.6)	4 (5.4)	
	T3	50	18 (36.0)	14 (28.0)	12 (24.0)	6 (12.0)	
Lymph node status	Positive	155	45 (29.0)	76 (49.0)	24 (15.5)	10 (6.5)	0.015/0.044 (0.21)
	Negative	76	13 (17.1)	32 (42.1)	24 (31.6)	7 (9.2)	
Histologic grade	I	105	31 (29.5)	49 (46.7)	20 (19.0)	5 (4.8)	0.015/0.044 (0.19)
	II	83	20 (24.1)	45 (54.2)	12 (14.5)	6 (7.2)	
	III	43	7 (16.3)	14 (32.6)	16 (37.2)	6 (14.0)	
Molecular subtype	Luminal	137	32 (23.4)	82 (59.9)	18 (13.1)	5 (3.6)	<0.001/<0.001 (0.29)
	HER2-positive	52	11 (21.2)	16 (30.8)	21 (40.4)	4 (7.7)	
	TNBC	42	15 (35.7)	10 (23.8)	9 (21.4)	8 (19.0)	
Total		231	58 (25.1)	108 (46.8)	48 (20.8)	17 (7.4)	

Values are n (row %). Unadjusted *p*-values were obtained using the Fisher–Freeman–Halton exact test (Monte Carlo, 50,000 samples); effect size is given as Cramér’s V. Holm-adjusted *p*-values account for the four primary comparisons. Unadjusted linear-by-linear (ordinal trend) *p*-values: histologic grade 0.004, lymph node status 0.006, tumour size 0.63. Luminal, ER- and/or PR-positive, HER2-negative; HER2-positive, ER- and PR-negative, HER2-positive; TNBC, triple-negative breast cancer.

## Data Availability

The data presented in this study are available on request from the corresponding author.
